# Long-Term Safety and Efficacy of Pars Plana Vitrectomy for Uveitis: Experience of a Tertiary Referral Centre in the United Kingdom

**DOI:** 10.3390/jcm12093252

**Published:** 2023-05-02

**Authors:** Muhannd El Faouri, Naseer Ally, Myrta Lippera, Siddharth Subramani, George Moussa, Tsveta Ivanova, Niall Patton, Felipe Dhawahir-Scala, Carlos Rocha-de-Lossada, Mariantonia Ferrara, Assad Jalil

**Affiliations:** 1 Manchester Royal Eye Hospital, Oxford Road, Manchester M13 9WL, UK; muhannad_fa3ouri@hotmail.com (M.E.F.); george.moussa@nhs.net (G.M.); tsveta.ivanova@mft.nhs.uk (T.I.); niall.patton@mft.nhs.uk (N.P.); mariantonia.ferrara@gmail.com (M.F.); 2 Faculty of Medicine, The Hashemite University, P.O. Box 330127, Zarqa 13133, Jordan; 3 Khoo Teck Puat Hospital, Singapore 768828, Singapore; 4 Qvision, Opththalmology Department, VITHAS Almería Hospital, 04120 Almería, Spain; 5 Ophthalmology Department, VITHAS Málaga, 29016 Málaga, Spain; 6 Regional Universityu Hospital of Malaga, Plaza del Hospital Civil, 29010 Málaga, Spain; 7 Surgery Department, University of Sevilla, 41009 Seville, Spain; 8 School of Medicine, University of Málaga, 29016 Málaga, Spain

**Keywords:** intraocular inflammation, pars plana vitrectomy, uveitis, vitreous debris, visual acuity, vitreous opacities

## Abstract

Aim: To evaluate the effectiveness of pars plana vitrectomy (PPV) without macular intervention on uveitis eyes with persistent vitreous inflammation/opacities in terms of visual acuity (VA), intraocular inflammation and macular profile. Methods: We carried out a single-center retrospective study of patients with uveitic eyes that underwent PPV without intervention on the macula due to persistent vitreous inflammation/opacities. The primary outcome measures were best-corrected visual acuity (BCVA), intraocular inflammation and macular profile at 3, 12 and 24 months after surgery. Results: Twenty-seven eyes of twenty-six patients were analyzed. Overall, 77.8% had an improvement of VA (55% by 0.3 LogMAR or more); 62.5% of patients had no intraocular inflammation, and the number of patients on systemic steroids and second-line immunosuppressives was reduced by 26% at 12 months; 87.5% of patients had resolution of macular oedema at 12 months. Conclusion: PPV for persistent vitreous inflammation/opacities is safe and effective, showing beneficial outcomes in terms of improvement of BCVA and the reduction in inflammation.

## 1. Introduction

Uveitis is a heterogenous group of pathological conditions characterized by intraocular inflammation and is associated with a wide variety of etiologies [1]. Both adults and, less commonly, pediatric patients can be affected by uveitis, and the consequences of a protracted or recurrent inflammation can be severe, potentially leading to significant visual impairment [2,3,4]. In particular, uveitis can cause vision loss in up to 20% of eyes, with chronic macular oedema being the main reason for a moderate level of vision loss, and macular scarring being the most common cause of irreversible severe vision loss [3]. Pars plana vitrectomy (PPV) currently represents one of the treatment options, and it is increasingly used to treat uveitis for diagnostic and/or therapeutic purposes [1]. Indeed, there can be multiple indications for performing PPV to treat uveitic eyes. First, large amounts of vitreous material can be obtained for microbiological and histopathological analyses to help identify the etiology in cases of atypical clinical presentation or history, inconclusive laboratory or radiologic testing or persistent inflammation or appropriate immunosuppression [5]. Second, PPV has a primary role in the management of posterior segment complications associated with uveitis, including vitreous hemorrhage, epiretinal membrane (ERM), full-thickness macular holes, retinal detachment (RD) and cyclitic membranes causing hypotony [6,7]. In addition, although its exact role as an anti-inflammatory therapy for uveitis remains uncertain, PPV has shown to be effective in improving visual acuity, intraocular inflammation and macular oedema and causing a reduction in immunosuppressive medications [8]. It has been speculated that the therapeutic effect of PPV may be due to the removal of inflammatory cells, cytokines and immune complexes from the vitreous body [8]. However, to date, therapeutic PPV has not been integrated in the routine management of uveitis, mainly due to the lack of specific indications and concerns regarding the potential complications of PPV in eyes with active inflammation and the stability of long-term outcomes [9,10]. 

In light of this background, the aim of our study was to evaluate the long-term safety and efficacy of PPV given to patients with uveitic eyes in terms of the control of intraocular inflammation, visual acuity and the macular profile. Specifically, in order to better assess the role of the PPV itself and exclude the potential bias associated with additional surgical maneuvers, such as ERM peeling, we focused on eyes where persistent vitreous involvement was the only indication for surgery, and no macular intervention was performed.

## 2. Materials and Methods

We conducted a retrospective, single-center, non-randomized interventional study of consecutive patients affected by uveitis with persistent vitreous inflammation/opacities who underwent PPV without intervention on their macula at the Manchester Royal Eye Hospital. The study was registered with the clinical audit department, and the research adhered to the tenets of the Declaration of Helsinki. Approved patient consent was obtained before all procedures and all surgeries were performed prior to the study design.

### 2.1. Participants and Data Collection

Data were identified and collected from the surgical database of the vitreoretinal unit and were anonymized for the analysis. We included eyes treated with PPV due to persistent vitreous inflammation/opacities associated with uveitis and a minimum follow-up (FU) of 24 months. Diagnosis of uveitis etiology was based on the patients’ history, clinical examination and investigations such as blood tests, chest radiographs and vitreous sampling (if performed) at the time of vitrectomy. The exclusion criteria were: (i) having undergone a surgical procedure on the macula (peeling of the ERM and/or internal limiting membrane); (ii) having a reason for being treated with PPV other than persistent vitreous inflammation/opacities, such as ERM, retinal detachment, a full-thickness macular hole and dislocated intraocular lens (IOL); (iii) having eyes where the vitreous sample was diagnostic for a non-inflammatory disease, specifically vitreoretinal lymphoma; (iv) patients with an incomplete FU.

Data collection included the patients’ demographics, the type of uveitis, indication for PPV, surgical details, therapeutic regimen and preoperative clinical findings and 3, 12 and 24 months after the surgery. At each follow-up, the ophthalmic examination included an assessment of best-corrected visual acuity (BCVA) expressed as a logarithm of the minimal angle of resolution (LogMAR) and intraocular pressure (IOP), a slit lamp examination, a dilated fundus examination and optical coherence tomography (OCT) of the macula. Uveitis activity was defined based on anterior chamber (AC) and graded by applying the SUN criteria [11], and vitreous inflammation was graded according to Nussenblatt’s method [12]. In particular, uveitis activity was defined as “mild”, “moderate” or “severe” if AC and/or vitreous inflammation values were ≤+1, +2 or +3, and +4, respectively. The presence of cystoid macular edema (CME) and central retinal thickness were recorded by analyzing macular OCT scans. The therapeutic regimen was defined as being given: (i) no medication, (ii) a topical steroid (TS), (iii) an oral steroid (OS), (iv) conventional or biologic Disease Modifying Anti-Rheumatic Drugs (DMARDs) or (v) a combination of them.

### 2.2. Surgical Procedure

All surgical procedures were performed by a consultant surgeon or a vitreoretinal fellow under local anesthesia. Three-port standard PPV (23, 25 or 27 gauge) was carried out using the Stellaris vitrectomy system (Bausch and Lomb, St. Louis, MO, USA) or Constellation vitrectomy system (Alcon Laboratories, Inc, Fort Worth, TX, USA). Combined cataract surgery with intraocular lens implantation was performed if lens’ opacity was detected preoperatively. Posterior vitreous detachment was induced, if it was needed, and core vitrectomy and peripheral vitreous shaving were performed. A vitreous sample was sent to the histopathological department for analysis. After the peripheral retinal check to look for retinal breaks, air or gas was used as an intraocular tamponade. All patients were given topical steroids, cycloplegia and antibiotics postoperatively. The topical steroid dose and, if necessary, oral steroid therapy was adjusted perioperatively to control inflammation.

### 2.3. Statistical Analysis

All data were analyzed using Stata 16.1 (Statacorp, College Station Texas, USA). Means (standard deviation, SD) were used to represent demographic data that had a normal distribution, and medians (Interquartile ranges) were used for skewed data. Crude analysis of paired skewed data was carried out using the Wilcoxon sign rank test. Due to the retrospective nature of the study and the presence of missing data at some time points, multiple imputation using chained equations was used to account for the missing data. As this study was a longitudinal analysis of the four main outcomes at four consecutive time periods, multi-level mixed effects models were used to account for the covariance of the data at each time point. Specifically, BCVA and CRT were assessed using mixed effects linear regression models. Uveitis activity and the presence of CME were assessed using a mixed effects ordinal logistic regression and mixed effects logistic regression models, respectively. All three FU visits were compared to the baseline. Other independent variables of clinical importance were added to generate the final multivariate models. Graphs of means were generated for time series data, with 95% confidence intervals represented. A *p*-value of <0.05 was regarded as significant.

## 3. Results

**Figure 1:**
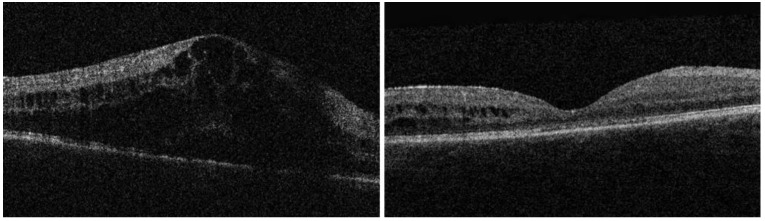
Case 1: Eye with vitritis and cystoid macular edema at the baseline (**left**); at 24-month, cystoid macular edema significantly improved, with persistence of small intraretinal cysts temporally involving the outer and inner nuclear layers (**right**).

**Figure 2:**
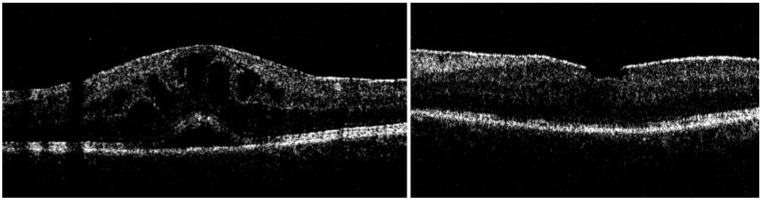
Case 2: Eye with vitritis and cystoid macular edema at the baseline (**left**); at 24-month, resolved cystoid macular edema and epiretinal membrane with preserved retinal layers (**right**).

A total of 27 eyes of 26 patients met the inclusion criteria and underwent PPV for persistent uveitis-related vitreous inflammation/opacities and were included in the analysis. The baseline characteristics are presented in Table 1. The mean (SD) age at the time of vitrectomy was 45.8 (18.7) years, with the range being from 8 to 86 years. Eight eyes underwent combined phaco-PPV, whereas the remaining ones were treated with vitrectomy only. Six eyes underwent cataract surgery during the FU period analyzed. In terms of ocular co-pathologies potentially affecting the visual outcomes at the final FU, we documented cases of CME (two eyes), ERM (two eyes), CME and ERM (two eyes), a macular scar (one eye) and a cataract (one eye) (Figure 1 and Figure 2). In addition, out of 27 eye, 6 eyes underwent glaucoma surgery during the FU (3 were treated with trabeculectomy and 3 was treated with glaucoma drainage implantation).

**Figure 3:**
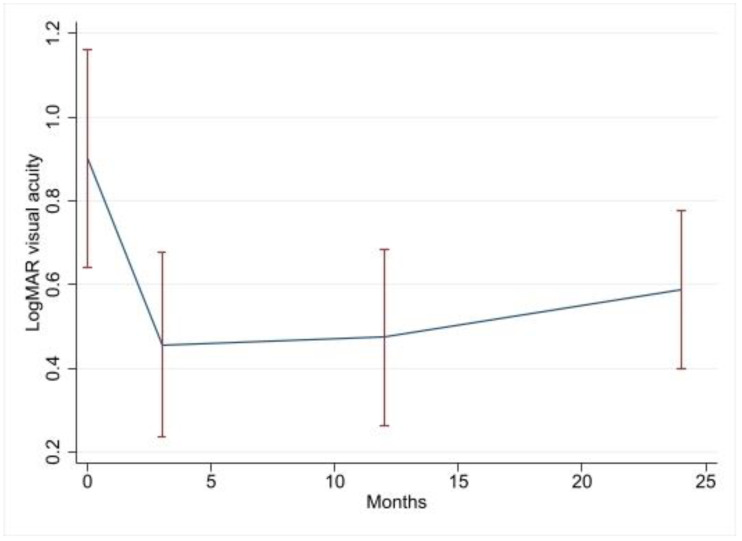
Time series plot of visual acuity recovery after pars plana vitrectomy in eyes of uveitic patients.

Overall, the crude change in BCVA was significant across all the follow-ups. The median (interquartile range, IQR) BCVA significantly improved from the preoperative value of 0.7 (0.50–1.00) logMAR to 0.30 (0.20–0.50) logMAR at the 3-month follow-up (*p* < 0.001), 0.30 (0.14–0.60) logMAR at the 12-month FU (*p* = 0.005) and 0.40 (0.10–0.60) logMAR at the 24-month FU (*p* = 0.003). Of the sample analyzed, 77.8% (*n* = 21) experienced improvement in BCVA, 11.1 % (*n* = 3) remained stable and 11.1.% (*n* = 3) worsened. The change in visual acuity outcomes over the study period are presented in Table 2 below. Univariate analysis showed that compared to the baseline, the visual acuity of patients improved by 0.45, 0.43 and 0.36 at 3, 12 and 24 months, respectively. The most significant improvement occurred in the period after the vitrectomy, and it slowly tapered off as the study period elapsed. Age and the presence of CME were also significant predictors. Every decade increase in age resulted in a 0.07 logMAR deterioration in visual acuity over two years. The presence of CME resulted in a 0.41 logMAR decrease in visual acuity if it was present over the entire study period. The pattern of visual recovery was maintained in the multivariate analysis, with patients improving by 0.43, 0.37 and 0.29 logMAR, respectively, over the three FU visits. Age, however, did not retain significance in the multivariate model, but the presence of CME did. The type of uveitis based on anatomical classification appears to have had an impact on the functional results, with intermediate uveitis showing a postoperative value of 0.35 logMAR, which is a better BCVA than that of posterior uveitis (*p* = 0.01). No significant difference was found when intermediate and panuveitis cases were compared, although there was a trend towards better results in the former group (*p* = 0.097). The combination of cataract surgery and PPV was significantly associated with postoperative BCVA as per univariate analysis (*p* = 0.014), but this association lost significance in the multivariate analysis (*p* = 0.21). Figure 3 below is a time series plot of the mean visual acuity with a 95% confidence interval at the baseline and each of the follow-up visits.

**Figure 4:**
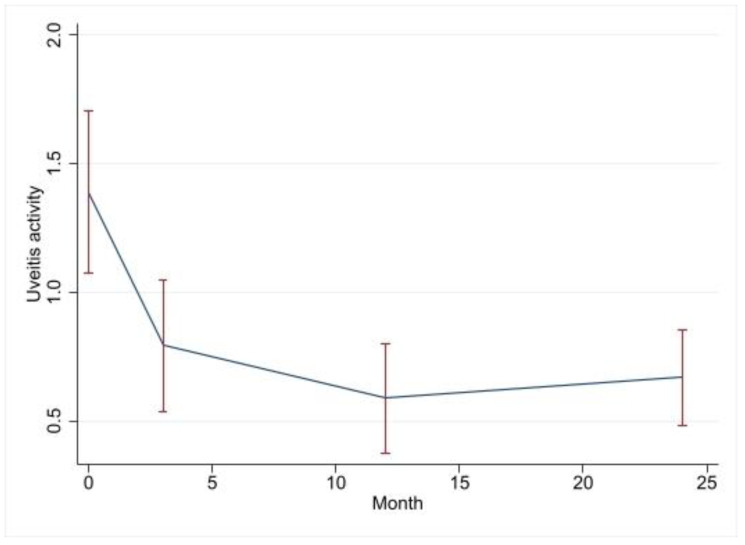
Change in uveitis activity at each follow-up visit in post-vitrectomized eyes.

The crude analysis of uveitis activity revealed that at the baseline, 7.4% (*n* = 2), 48.1% (*n* = 13), 37.0% (*n* = 10) and 7.4% (*n* = 2) patients had no activity and mild, moderate and severe activity, respectively. This improved to 44.4% (*n* = 12), 44.4% (*n* = 12), 3.7% (*n* = 1) and 3.7% (*n* = 1) with no activity and mild, moderate and severe activity at 3 months, respectively (*p* = 0.003). At the 12-month FU, the proportions of patients with no activity and mild, moderate and severe activity were 51.9% (*n* = 14), 40.7% (*n* = 11), 3.7% (*n* = 1) and 0%, respectively (*p* < 0.001). At the 24-month FU, the proportions of patients with no activity and mild, moderate and severe activity were 48.1% (*n* = 13), 40.7% (*n* = 11), 3.7% (*n* = 1) and 0%, respectively (*p* = 0.002). Table 3 below shows the change in uveitis activity at each of the follow-up visits compared to that of the baseline. The multivariate model shows a highly significant temporal relationship with uveitis grading, which decreased by 1.97, 2.28 and 2.19 at 3, 12 and 24 months, respectively (Table 4). Age was not a significant predictor of uveitis activity. Uveitis type was associated with the uveitis activity, with intermediate uveitis showing better response than panuveitis did (*p* = 0.002), but not posterior uveitis (*p* = 0.834). Figure 4 below shows the uveitis activity at each FU visit. As expected, due to the changes in uveitis activity, the therapeutic regimen was different at different FU visits. The therapeutic regimes at each FU are detailed in Table 3. In particular, at the baseline, 12 patients were on oral steroids, which was compared to 4 at the final FU.

**Figure 5:**
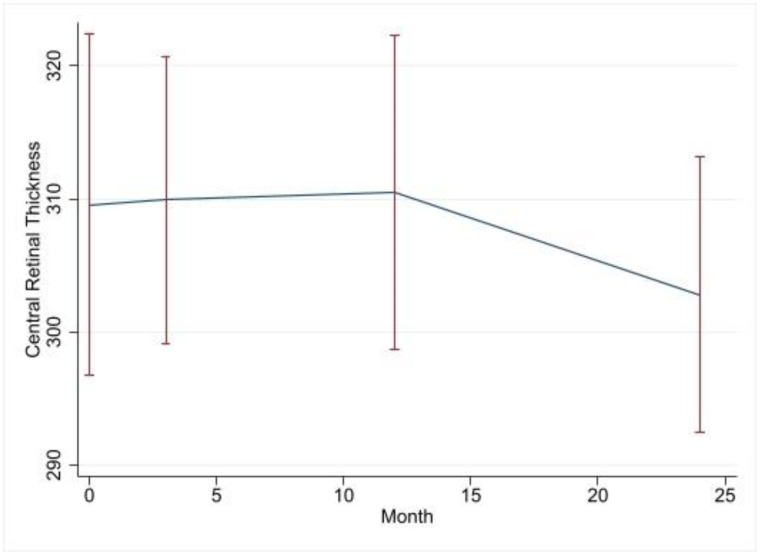
Time series plot of CRTs at baseline and each follow-up visit.

The mean CRT values at the baseline, 3, 12 and 24 months were 360 (147.1), 339 (77.9), 284 (41.9) and 245 (30.4), respectively. Table 5 below shows a non-significant change over time in CRT. There is, however, a trend towards significance with each follow-up visit, with the upper limit of the 95% confidence interval at 24 months being only 5.9 µm thicker than the baseline measurement is, while the lower limit is 119.7 µm thinner than the baseline measurement is. CME was a significant predictor of CRT, with patients with CME having a 78.5 µm thicker CRTs over the study period. Figure 5 illustrates the CRTs at the baseline and each follow-up visit.

We also specifically focused on the presence or absence of CME. At the baseline, 3, 12 and 24 months, 25.9% (*n* = 7), 14.8% (*n* = 4), 11.1% (*n* = 3), and 11.1% (*n* = 3) of patients had CME. The odds of developing CME over the study period did not significantly change when it was compared to that of the baseline as shown in Table 6 below.

## 4. Discussion

This study evaluated the long-term safety and efficacy of PPV for therapeutic and/or diagnostic purposes via persistent vitreous involvement in uveitic eyes, focusing on the postoperative control of intraocular inflammation, visual acuity and the macular profile. The rationale for including these patients relies on the attempt to selectively assess the potential effect of PPV itself and avoid confounding factors, in particular, concomitant pathologies. Indeed, the therapeutic role of PPV in uveitis has been variously reported as being beneficial for treating several diseases of the posterior segment affecting uveitic eyes, such as ERM, retinal detachment and persistent CME [6,7]. Our study demonstrated a beneficial effect of PPV in terms of BCVA, uveitis activity and the macular profile.

The improvement in visual acuity after the vitrectomy of uveitic eyes has been variously reported over recent decades [1,10]. Becker et al. reported 44 case series published between 1981 and 2005 and documented an improvement in vision in 708 eyes (68%) [8]. Shin et al. [9] analyzed the 12-month outcomes after PPV among patients with intermediate uveitis and reported a VA improvement in 69.7% of eyes, with mean preoperative BCVA improving from 0.81 (0.64) logMAR to 0.41 (0.50) logMAR postoperatively. Branson et al. [13] reviewed 11 studies reporting variable visual outcomes of PPV for macular pathology in uveitis, but this may be due to the masking of chronic permanent macular damage due to ERM or macular holes. Indeed, positive functional outcomes were further supported by a recent review reporting that the postoperative visual acuity improved in 69% of the eyes included, whereas no change and worsening were documented in 18% and 13%, respectively [10]. Our results appear to be consistent with previous studies, as the BCVA significantly improved at each FU visit. Interestingly, we report, for the first time, that although the BCVA improvement remained significant at 24-month FU, there was a significant trend towards a worsening of visual acuity with time. This finding may be explained by the presence of concomitant/subsequent ocular pathologies, such as glaucoma, ERM, cataract or posterior capsule opacification.

The functional improvement of eyes with uveitis after PPV has been also associated with the reportedly improved intraocular inflammation [9,14,15,16,17,18,19,20]. The effect of PPV on the inflammation status of eyes with uveitis has been evaluated in several previous studies, and the clearance of active inflammatory mediators from the vitreous cavity has been advocated as the main factor responsible for the decreased uveitis activity. However, other factors may contribute to the positive impact of PPV on intraocular inflammation, such as the increased clearance of inflammatory cells and the enhanced penetration of drugs used to control inflammation in vitrectomized eyes, as well as changes in the intraocular gradients of oxygen [21]. Conversely, in our study that included uveitis with different etiologies and anatomical location, most of previous studies focused on a specific type of uveitis. Shin et al. [9] studied the long-term implications of PPV performed in cases of complications, such as ERM, tractional retinal detachment and vitreous opacity, in 66 eyes with intermediate uveitis. The study demonstrated that in eyes treated with PPV, there was a decrease in the frequency of uveitis attacks, a reduction in the need for further therapy such as peri- or intraocular steroid injections or systemic medications, and an improvement in visual acuity during the 12-month follow up, which was independent of the degree of preoperative inflammation. Giuliari et al. [18] showed that PPV is safe and effective for the management of chronic pediatric uveitis and is associated with the reduction in systemic anti-inflammatory medications. Interestingly, Quinones et al. [22] performed a small randomized trial of 18 eyes, reporting more the improved control of intermediate uveitis after PPV compared with that of immunomodulatory therapy. Similar to our study, Scott et al. [15] included 41 eyes with intermediate cases, posterior cases or panuveitis treated with PPV, reporting an overall improvement in inflammation and visual acuity at the 12-month FU. Despite the better control of inflammation, the use of oral steroids and DMARDs remained unchanged in this cohort [15]. Takayama et al. [20] compared 38 eyes with granulomatous uveitis and 17 eyes with non-granulomatous uveitis treated with PPV and showed improvement in visual acuity and inflammation status 6 months after PPV. However, in the latter series, there was no analysis of the immunosuppressive and corticosteroid therapy [20]. Consistent with previous reports, we found a positive effect of PPV in terms of uveitis activity, which was significantly reduced at each FU analyzed. In terms of therapeutic regimen, we followed a step-wise approach, increasing the treatment from topical to oral steroids to determine the minimum amount of second-line immunosuppressives needed to achieve adequate control of intraocular inflammation. In addition, preoperative steroid and immunosuppressive therapy was avoided if PPV was performed for diagnostic purposes in order to improve the diagnostic yield. Improved inflammation control was also confirmed by the reduced proportion of patients requiring oral steroids 12 and 24 months after PPV. It is important to note that the control of inflammation without the need to use oral steroids is the main aim in the management of uveitis due to the potential steroid side effects, especially among children. 

Our study also supported the safety of PPV in uveitic eyes. Indeed, we reported a limited number of intraoperative and postoperative complications. In this regard, the dramatic advances of vitrectomy machines over the last two decades, resulting in a less invasive and more efficient procedure [23,24,25], have been advocated as a significant contributing factor to the wide use of PPV, even for eyes with uveitis.

The improved control of inflammation post-PPV may contribute to the reported beneficial effect on CRT and CME. Macular edema is defined as the thickening of the retina in the macula caused by a breakdown in the blood–retinal barrier (BRB) with an accumulation of extracellular fluid in the intra or sub-retinal area [26]. The uveitic ME can range from 20% to 70% and can occur as a complication in anterior, intermediate or posterior uveitis. It is frequently observed in chronic and intermediate or posterior uveitis and panuveitis [27,28]. Macular oedema is the major cause of visual loss in uveitis, causing a visual acuity of 20/60 or less in approximately 40% of eyes [26,27,29]. Macular edema can occur in uveitis with different patterns: cystoid ME, in up to 80% of the cases, diffuse ME and serous retinal detachment [28]. The pathogenesis of ME in uveitis is multi-factorial, and although mechanical vitreomacular interactions and membrane formation on the macular surface can play a role, persistent subclinical or chronic inflammation is a key factor in the process [26]. Indeed, inflammation causes retinal pigment epithelial pump dysfunction and the consequent breakdown of the blood–retina barrier with the leakage of fluid in the retinal tissue [26,28]. The accumulation of inflammatory cytokines in the vitreous, which acts as a depot, could also be partly responsible for the macular oedema [8,30]. Therefore, performing PPV to remove the vitreous can theoretically eliminate the accumulation of inflammatory mediators close to the macula and help resolve or reduce the mechanical vitreomacular interactions [8]. In our study, only seven patients had preoperative macular oedemas. Considering that macular oedemas are a common complication of uveitis [28], the occurrence of macular oedemas in our study is limited. One of the factors could be due to under-reporting and obscuration of the macula from significant vitreous debris in the patients. Furthermore, we excluded all PPV with surgical maneuvers performed on the macula, including internal limiting membrane peeling, which has been proposed as a therapeutic option when the persistent CME is the main indication for PPV [31,32,33]. In addition, the impact of ILM peeling in severe CME of different etiologies is still controversial [32,33,34]. Although PPV was not specifically used in any of our cases to treat a macular oedema, we reported the resolution of macular edemas in four of seven patients (57%). This is better than reports elsewhere with rates with a range of 40–60% [8,14,15,31,35,36]. The true percentage of improvement, and hence, the protective effect on the macula may potentially be greater if it is assumed that more patients may have had an undiagnosed macular oedema prior to PPV due to hazy media. In addition, we found a trend towards the reduction in CRT during the entire FU, further supporting the long-term beneficial effect of PPV on the macular profile. These results, along with the risk of irreversible macular damage associated with undiagnosed chronic CME, might encourage early PPV in uveitic eyes with persistent vitreous inflammation/opacities. In addition, the removal of inflammatory debris with the secondary reduction of the macular oedema following PPV may allow the safe tapering of immunosuppressive medications.

We acknowledge that this study has several limitations. Firstly, the retrospective design will inherently lead to case selection bias. Moreover, due to the small sample size, we did not subgroup the uveitis conditions based on etiology, as this would have made the group sizes very small and provided inconclusive results. However, it should be highlighted that uveitis is not a common condition, and the application of strict inclusion and exclusion criteria allowed us to present a more homogeneous sample and exclude potential biases associated with specific surgical maneuvers (e.g., ERM peeling) or other main surgical indications (e.g., RD).

In conclusion, we report one of the largest studies on uveitic vitrectomies looking at both the control of inflammation and the use of immunosuppressive medications at 24 months following the surgery for all types of uveitis irrespective of the cause. Our results have shown that PPV, even without macular intervention for uveitis, leads to an overall improvement in VA, macular oedema and intraocular inflammation. Furthermore, there is an overall reduction in the use of combination medical therapy to control intraocular inflammation. The development of a well-designed randomized controlled trial for PPV in improving outcomes for uveitis will be useful, but it remains a challenge to conduct this type of study due to the heterogenous causes of uveitis.

## Figures and Tables

**Table 1:** Baseline characteristics of study patients and eyes that underwent vitrectomy.

Baseline Characteristic	
Age (years) mean (SD)	45.8 (18.7)
Sex, male *n* (%)	16 (59.3)
Lens status—*n* (%)	Phakic without cataract—11 (40.8) Phakic with cataract—8 (29.6) Pseudophakic—8 (29.6)
Uveitis etiology—*n* (%)	Idiopathic—4 (14.8) Sarcoidosis—9 (33) Toxoplasma—5 (18.5) Tuberculosis—1 (3.7) Fuchs Uveitis Syndrome—6 (22.2) Varicella-zoster virus—primary/active—1 (3.7) Toxocara—1 (3.7)
SUN classification—*n* (%)	Intermediate—9 (33) Posterior—9 (33) Panuveitis—9 (33)
Primary indication for vitrectomy—*n* (%)	Clearance of inflammatory debris—20 (74.1) Clearance of Hemorrhagic debris—3 (11.1) Cytological sampling—4 (14.8)

**Table 2:** Multi-level mixed effects linear regression model of visual acuity outcome.

Variable	Change in LogMAR Visual Acuity Compared to Baseline	95% CI	*p* Value
Univariate			
3-month visit	−0.45	−0.76–−0.13	0.006
12-month visit	−0.43	−0.75–−0.11	0.008
24-month visit	−0.36	−0.69–−0.04	0.027
Age (Decade)	0.07	0.001–0.013	0.027
CME	0.41	0.15–0.68	0.002
Posterior compared to Intermediate	0.56	0.29–0.84	<0.001
Panuveitis compared to Intermediate	0.15	−0.13–0.42	0.29
Baseline Pseudophakic compared to phakic	−0.23	−0.51–0.04	0.098
Combined phacovitrectomy	0.32	0.07–0.58	0.014
Multivariate			
3-month visit	−0.46	−0.74–−0.18	0.002
12-month visit	−0.39	−0.67–−0.11	0.007
24-month visist	−0.30	−0.59–−0.013	0.041
Age (Decade)	0.04	−0.002–0.011	0.17
CME	0.36	0.11–0.62	0.005
Posterior compared to Intermediate	0.35	0.08–0.61	0.01
Panuveitis compared to Intermediate	0.21	−0.04–0.45	0.097
Baseline pseudophakic compared to phakic *	−0.21	−0.45–0.04	0.093
Combined phacovitrectomy *	0.16	−0.09–0.40	0.21

* Baseline lens status and combined phacovitrectomy were assessed separately in the multivariate models. Final reported coefficients were based on visit, CMO and diagnosis data.

**Table 3:** Therapeutic regimen during the follow-up.

Therapeutic Regimen	Baseline Eyes, *n* (%)	3 m FU Eyes, *n* (%)	12 m FU Eyes, *n* (%)	24 m FU Eyes, *n* (%)
None	7 (25.9)	2 (7.4)	5 (18.5)	6 (22.2)
TS	8 (29.6)	19 (70.4)	15 (55.6)	15 (55.6)
OS	2 (7.4)	0 (0)	0 (0)	0 (0)
TS + OS	7 (25.9)	4 (14.8)	5 (18.5)	2 (7.4)
TS + DMARD	0	1 (3.7)	1 (3.7)	1 (3.7)
TS + OS + DMARD	3 (11.1)	1 (3.7)	1 (3.7)	2 (7.4)

**Table 4:** Multi-level mixed effects ordered logistic regression model of uveitis activity.

Variable	Change in Uveitis Activity Compared to that of the Baseline	95% CI	*p* Value
3-month visit	−2.14	−3.3–−1.00	<0.001
12-month visit	−2.50	−3.66–−1.33	<0.001
24-month visit	−2.34	−3.5–−1.16	<0.001
Age	0.005	−0.02–−0.03	0.625
Posterior compared to Intermediate	−0.11	−1.14–0.92	0.834
Panuveitis compared to Intermediate	1.53	0.56–2.51	0.002

**Table 5:** Multilevel mixed effects linear regression model.

Variable	Change in Central Retinal Thickness Compared to that of the Baseline	95% CI	*p* Value
3-month visit	−7.8	−71.2–55.6	0.809
12-month visit	−34.4	−101.5–32.6	0.312
24-month visit	−56.9	−119.7–5.9	0.076
Age	−0.15	−1.7–−1.4	0.847
CME	78.5	26.0–131.0	0.003

**Table 6:** Multi-level mixed effects logistic regression model.

Variable	Odds Ratio of CME Compared to Baseline	95% CI	*p* Value
3-month visit	0.66	0.2–2.9	0.576
12-month visit	0.51	0.1–2.5	0.404
24-month visit	0.50	0.98–2.5	0.394
Age	0.99	0.96–1.03	0.834
CME	1.03	0.07–3.06	0.993

## Data Availability

The data that support the findings of this study are available from the corresponding author upon reasonable request.
